# Computed tomography-based radiomics quantification predicts epidermal growth factor receptor mutation status and efficacy of first-line targeted therapy in lung adenocarcinoma

**DOI:** 10.3389/fonc.2022.985284

**Published:** 2022-08-16

**Authors:** Meilin Jiang, Pei Yang, Jing Li, Wenying Peng, Xingxiang Pu, Bolin Chen, Jia Li, Jingyi Wang, Lin Wu

**Affiliations:** ^1^ The Second Department of Thoracic Oncology, Hunan Cancer Hospital, The Affiliated Cancer Hospital of Xiangya School of Medicine, Central South University, Changsha, China; ^2^ The General Surgery Department of Xiangya Hospital Affiliated to Central South University, Changsha, China; ^3^ The National Clinical Research Center for Geriatric Disorders of Xiangya Hospital Affiliated to Central South University, Changsha, China; ^4^ Medical Oncology, Hunan Cancer Hospital, The Affiliated Cancer Hospital of Xiangya School of Medicine, Central South University, Changsha, China; ^5^ The Second Department of Oncology, Yunnan Cancer Hospital, The Third Affiliated Hospital of Kunming Medical University, Yunnan Cancer Center, Kunming, China

**Keywords:** lung adenocarcinoma, computed tomography, radiomic response biomarker, epidermal growth factor receptor mutation status, machine learning

## Abstract

**Background:**

Biomarkers that predict the efficacy of first-line tyrosine kinase inhibitors (TKIs) are pivotal in epidermal growth factor receptor (EGFR) mutant advanced lung adenocarcinoma. Imaging-based biomarkers have attracted much attention in anticancer therapy. This study aims to use the machine learning method to distinguish EGFR mutation status and further explores the predictive role of EGFR mutation-related radiomics features in response to first-line TKIs.

**Methods:**

We retrospectively analyzed pretreatment CT images and clinical information from a cohort of lung adenocarcinomas. We entered the top-ranked features into a support vector machine (SVM) classifier to establish a radiomics signature that predicted EGFR mutation status. Furthermore, we identified the best response-related features based on EGFR mutant-related features in first-line TKI therapy patients. Then we test and validate the predictive effect of the best response-related features for progression-free survival (PFS).

**Results:**

Six hundred ninety-two patients were enrolled in building radiomics signatures. The 13 top-ranked features were input into an SVM classifier to establish the radiomics signature of the training cohort (n = 514), and the predictive score of the radiomics signature was assessed on an independent validation group with 178 patients and obtained an area under the curve (AUC) of 74.13%, an F1 score of 68.29%, a specificity of 79.55%, an accuracy of 70.79%, and a sensitivity of 62.22%. More importantly, the skewness-Low (≤0.882) or 10th percentile-Low group (≤21.132) had a superior partial response (PR) rate than the skewness-High or 10th percentile-High group (*p* < 0.01). Higher skewness (hazard ratio (HR) = 1.722, *p* = 0.001) was also found to be significantly associated with worse PFS.

**Conclusions:**

The radiomics signature can be used to predict EGFR mutation status. Skewness may contribute to the stratification of disease progression in lung cancer patients treated with first-line TKIs.

## Introduction

Lung cancer is the most prevalent cancer worldwide, causing the highest cancer-related death rate of all malignancies (1). Adenocarcinoma comprises 80% of non-small cell lung cancer (NSCLC), and epidermal growth factor receptor (EGFR) mutations mostly appear in this subtype (2, 3). With the discovery and development of tyrosine kinase inhibitors (TKIs), the clinical treatment strategy for advanced activating EGFR mutation lung adenocarcinoma has evolved into a personalized approach (4, 5). Based on the National Comprehensive Cancer Network (NCCN) and Chinese Society of Clinical Oncology (CSCO) guidelines, EGFR TKIs have been approved as first-line standard therapy for driver mutation-positive metastatic adenocarcinoma based on studies that have shown better survival than chemotherapy (3, 6–9).

Nowadays, the individual diagnosis and treatment of EGFR-mutant lung adenocarcinoma depend on invasive biopsy testing. However, low DNA quality and testing methods can limit the reliability of results and sequencing applications (10–14). Furthermore, the EGFR mutation result was only determined by a part of tumor tissue, ignoring the heterogeneity of the entire tumor, which might be the reason for the inconsistent treatment outcome. When patients preliminarily elect for EGFR-TKI therapy only based on EGFR mutation, their response will not last long and varies so markedly after treatment (4, 9, 15). In sum, it is crucial and urgent to use the whole picture of the tumor to predict the potential resistance or the likelihood of rapid progression comprehensively before patients receive EGFR TKIs.

Radiomics is a non-invasive and high-throughput image assessment approach based on medical imaging (16, 17). A correlation between radiomics features and underlying intertumoral heterogeneity of lung cancer has been observed (18–26). Furthermore, molecular images have been used to identify patients with different therapeutic outcomes of EGFR-TKI therapy (27–30). Tian et al. built a signature to discriminate lung cancer patients with rapid and slow progression to EGFR-TKI therapy using the least absolute shrinkage and selection operator (LASSO) Cox regression model based on two-direction imaging data. Cook et al. found the association between features and survival by Cox regression analyses. However, compared to the predictive model that was made of an ‘unknown process’, oncologists tend to identify some specific image features and link them to the medical explanation.

Hence, our study aimed to locate some specific image features that were highly related to the survival outcome and could be linked to clinical practice. We proposed a radiomics signature based on all three computed tomography (CT) image dimensions for predicting EGFR mutation status. We further explored in-depth the relevance between EGFR mutation-related features and risk stratification of progression-free survival (PFS) in EGFR mutant advanced adenocarcinoma.

## Materials and methods

### Patients

**Figure 1:**
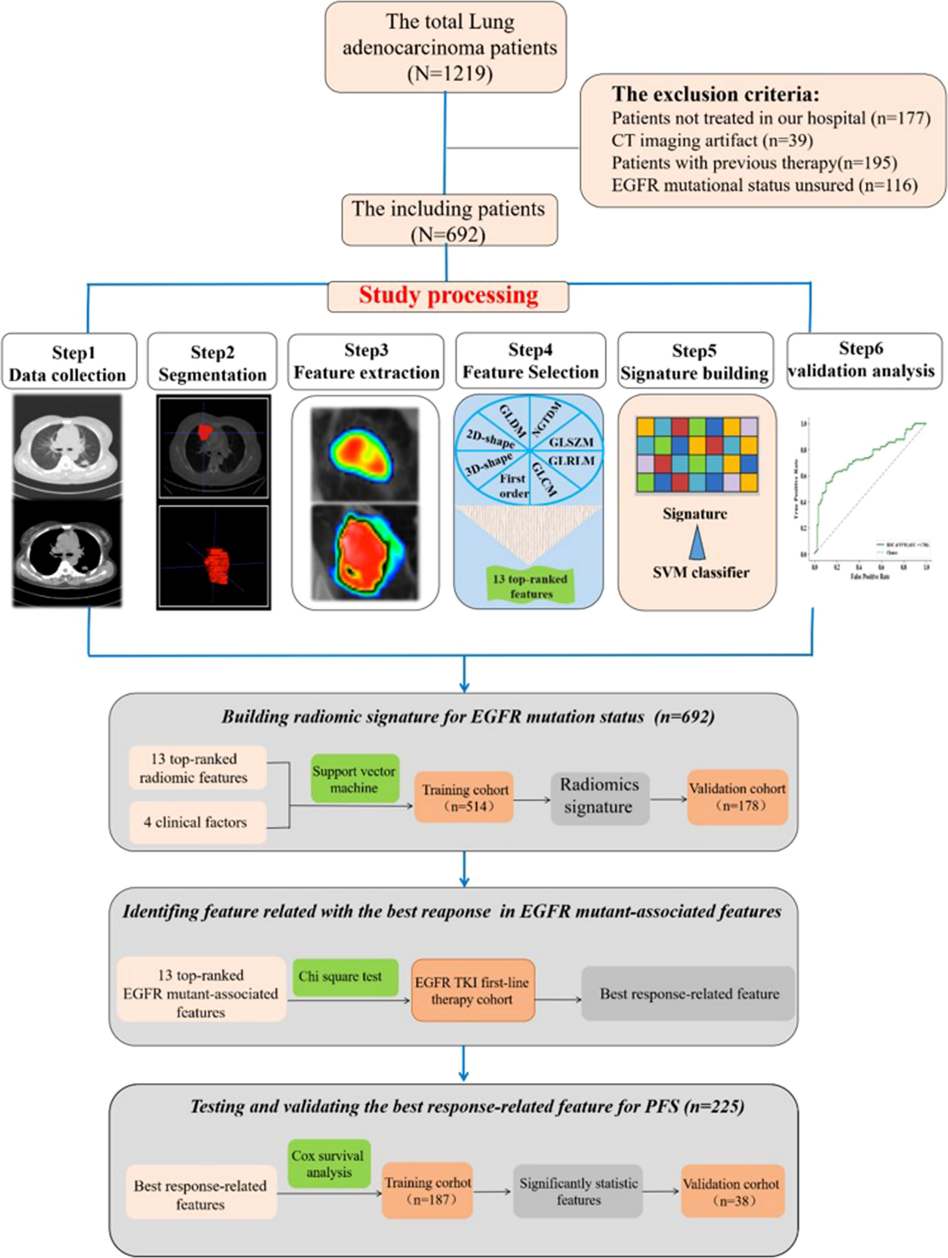
Images show study processing of radiomics. Computed tomography (CT) data were retrospectively collected. Region of interest was manually segmented in axial view by a clinical doctor using imaging biomarker explorer software. Eight categories of radiomics features were extracted from region of interest (ROI) in CT images and next, the top 13 features to train support vector machine classifier and validate it on independent set (n = 178). Experiment 1 is for developing radiomics signature for epidermal growth factor receptor (EGFR) mutational status in lung adenocarcinoma. Experiment 2 is for analyzing the relationship between progression-free survival and the top 13 features.

The institutional research board of Hunan Cancer Hospital (Changsha, China) approved this retrospective study. A total of 1,219 lung adenocarcinoma patients at Hunan Cancer Hospital were initially collected between July 2013 and September 2019. Patients were included in this research based on the following inclusion criteria: 1) pathologically confirmed primary pulmonary adenocarcinoma in our institute, 2) there are measurable target lesions under the Response Evaluation Criteria in Solid Tumors version 1.1 (RECIST v 1.1), 3) next-generation sequencing-proven EGFR mutational status by tumor tissue sample, and 4) available patient characteristic clinical data. Finally, 692 patients were included in our study. Furthermore, clinical data were collected, including therapy protocol, response evaluation, and follow-up material. In the process of building the predictive radiomics signature, patients confirmed between 1 July 2013 and 1 May 2018 were enrolled in a training cohort, and those confirmed between 1 June 2018 and 1 September 2019 were enrolled in a test cohort (**Figure 1**).

Response assessment routinely took place 4–6 weeks after treatment completion by diagnostic CT scans and laboratory tests according to the RECIST v 1.1. PFS is the study endpoint considered the time from the initiation of therapy to confirmation of progression or death.

### CT scanning protocol

All thoracic CT examinations were performed at Hunan Cancer Hospital. CT images of all patients were acquired on CT-on-rails (Brilliance CT 16, Hunan Tumor Hospital, Changsha) with the following parameters: a 5.0-mm slice thickness reconstruction, 313-mA tube current, and 120-kV peak voltage.

### Tumor imaging segmentation and feature extraction

In this study, all nodules were identified by a radiologist with more than 10 years of experience, and the clinician manually annotated the regions of interest (ROIs) on axial view piece by piece using imaging biomarker explorer (IBEX) software (31, 32). In the end, each ROI of the subject was reviewed by a radiologist. Imaging features were extracted by the PyRadiomics toolbox (33), which is an open-source python software package. To mine rich radiomics, each original image was processed by eight image filters. 1) Wavelet filter: yields eight decompositions per level (all possible combinations of applying either a high- or low-pass filter in each of the three dimensions). 2) Laplacian of Gaussian filter: edge enhancement filter, emphasizes areas of gray-level change, where sigma defines how coarse the emphasized texture should be. A low sigma emphasis on fine textures (change over a short distance), where a high sigma value emphasizes coarse textures (gray-level change over a large distance). 3) Square: takes the square of the image intensities and linearly scales them back to the original range. 4) SquareRoot: takes the square root of the absolute image intensities and scales them back to the original range. 5) Logarithm: takes the logarithm of the absolute intensity + 1. 6) Exponential: takes the exponential, where filtered intensity is e^(absolute intensity). 7) Gradient: returns the magnitude of the local gradient. 8) Local Binary Pattern: computes the Local Binary Pattern in a by-slice operation (two-dimensional (2D)) and three-dimensional (3D) using spherical harmonics (34). Then, the features were quantified by the following eight categories of imaging features: 1) first-order statistics with 19 features, 2) 3D shape-based with 16 features, 3) 2D shape-based with 10 features, 4) gray-level co-occurrence matrix (GLCM) with 24 features, 5) gray-level run length matrix (GLRLM) with 16 features, 6) gray-level size zone matrix (GLSZM) with 16 features, 7) neighboring gray-tone difference matrix (NGTDM) with five features, and 8) gray-level dependence matrix (GLDM) with 14 features. In the end, 2,153 quantitative radiological features from each ROI were obtained.

### Feature selection and signature building

The Mann–Whitney statistical test (13) was first conducted to distinguish the redundant features. Each feature with a *p*-value >0.05 was redundant and eliminated. After redundant features were removed, the residual parameters were normalized by the z-score method, which is widely used in machine learning. Then, the feature where the variance is equal to zero was removed again. To further decrease the dimension, the minimum redundancy maximum relevance (mRMR) method was used to determine the most remarkable radiomics features.

Finally, the top-ranked radiomics features were entered into a support vector machine (SVM) classifier to establish a radiomics signature that predicts EGFR mutation status. The parameters of the classifier were optimized by a grid searching technology on the training cohort using 10-fold cross-validation. The radiomics signature with the best accuracy was confirmed. Previous studies have shown that clinical features are associated with the outcome of lymph node metastasis (35). In this study, we found a radiomics signature based on the top-ranked features and then added critical clinical features to explore the predictive score of EGFR mutation status.

### Evaluation of radiomics signature

The performance of the radiomics signature in predicting EGFR mutation status was estimated by the area under the curve (AUC) of the receiver operating characteristic (ROC) curve. In addition, accuracy, sensitivity, specificity, and an F1 score were also used to measure the signature.

### Statistical analysis

Statistical analysis was performed with SPSS version 22. The independent-samples t-test was used to evaluate the difference in median age between the EGFR-positive and EGFR-negative groups. The chi-square test was used for statistical analysis of gender, tumor stage, smoking history, family history, and tumor position. In the EGFR mutational advanced patients, the cutoff points of statistically significant features were defined by the AUC value of the ROC curve. Survival analysis included patients with disease progression treated with first-line EGFR TKIs. Based on the cutoff points, the chi-square test was used to identify the relationship between radiomics features and the best response. Cox regression analysis was used to explore the predictive capability of the best response-related features for PFS. Parameters with a *p*-value <0.1 in univariate analysis were selected in multivariate Cox proportional hazards regression analysis. The results were presented as hazard ratio (HR) and 95% CI. The reported statistical remarkable levels were all two-sided, and *p*-values <0.05 were significant.

## Results

### Patient characteristics

The clinicopathologic features of patients are shown in **Table 1**. In all patients, 355 patients with confirmed EGFR-positive type were enrolled, while 337 patients were EGFR wild type. Most patients were diagnosed with inoperable stage III or IV disease (677/692, 97.8%), and 50.4% of 692 patients were former or active smokers (**Table 1**). Patient characteristics including age, gender, and smoking history were demonstrated to be different between EGFR-positive and EGFR-negative type cohorts, which is consistent with a previous clinical study (**Table 1**).

**Table 1:** Clinical characteristics of all patients included in the study.

Factors	Training cohort		*p*-Value	Validation cohort		*p*-Value
	EGFR-wild	EGFR-mutant		EGFR-wild	EGFR-mutant	
Subject (*N*)	514			178		
Age (years)	57 ± 9	55 ± 4	<0.001^b^	57 ± 11	59 ± 9	<0.001^b^
Gender			<0.001^b^			<0.001^b^
Male	171	136		76	40	
Female	78	129	12	50
Smoking history			<0.001^b^			<0.001^b^
Yes	158	95		67	29	
No	91	170	21	61
Family history			0.417			0.565
Yes	31	26		11	15	
No	218	239	77	75
TNM stage^a^			0.717			0.076
I	2	2		0	1	
II	5	3	2	0
III	42	38	11	4
IV	200	222	75	85
Tumor position			0.853			0.116
RUL	81	87		18	27	
RML	20	27	12	16
RLL	45	52	14	16
LUL	66	63	17	18
LLL	37	36	27	13
EGFR mutation type	0	265		0	90	
Wild type	249	0		88	0	
Exon 19 deletion	0	167	0	58
Exon 21 insertion	0	89	0	31
Other types	0	9	0	1

EGFR, epidermal growth factor receptor; RUL, right upper lung; RML, right middle lung; RLL, right lower lung; LUL, left upper lung; LLL, left lower lung.

a Based on American Joint Committee on Cancer (AJCC) 8th edition.

b Only statistically significant (p < 0.05) results are reported for analysis.

Two hundred twenty-five patients with EGFR mutation who experienced disease progression following first-line TKI therapy were included in the efficacy analysis presented in **Table 2**. The median follow-up time was 1 year (range, 0.7–37.7 months). In the training and validation cohorts (187 and 38 cases, respectively), the results showed no significant difference in PFS (median PFS: training cohort, 12 months; validation cohort, 11.8 months; Mann–Whitney, *p* = 0.304). Moreover, there were also no significant differences (*p* > 0.05) regarding age, gender, smoking history, family history, tumor stage, and position between the two cohorts (**Table 2**).

**Table 2:** Clinical characteristics of patients included in treatment response analysis.

Factors	Training cohort	Validation cohort	*p*-Value
	N (%)	N (%)	
Subject(*N*)	187 (100)	38 (100)	
Age(years)			0.114
Median	55	57	
Range	29–80	37–75	
Gender			0.707
Male	80 (42.8)	15 (39.5)	
Female	107 (57.2)	23 (60.5)	
Smoking history			0.894
Yes	57 (30.5)	12 (31.6)	
No	130 (69.5)	26 (68.4)	
Family history			0.469
Yes	19 (10.2)	6 (15.8)	
No	168 (89.8)	32 (84.2)	
TNM stage^a^			0.083
III	19 (10.2)	0 (0)	
IV	168 (89.8)	38 (100)	
Tumor position			0.466
RUL	57 (30.5)	9 (23.7)	
RML	17 (9.1)	9 (23.7)	
RLL	33 (17.7)	9 (23.7)	
LUL	53 (28.3)	7 (18.4)	
LLL	27 (14.4)	4 (10.5)	
EGFR-TKI therapy			0.718
Gefitinib	66	13	
Erlotinib	62	15	
Icotinib	59	10	
Median PFS (months)	12	11.8	0.304

PFS, progression-free survival; RUL, right upper lung; RML, right middle lung; RLL, right lower lung; LUL, left upper lung; LLL, left lower lung; EGFR, epidermal growth factor receptor; TKI, tyrosine kinase inhibitor.

a Based on American Joint Committee on Cancer (AJCC) 8th edition.

### Building and validating the predictive radiomics signature for epidermal growth factor receptor mutation status

**Figure 2:**
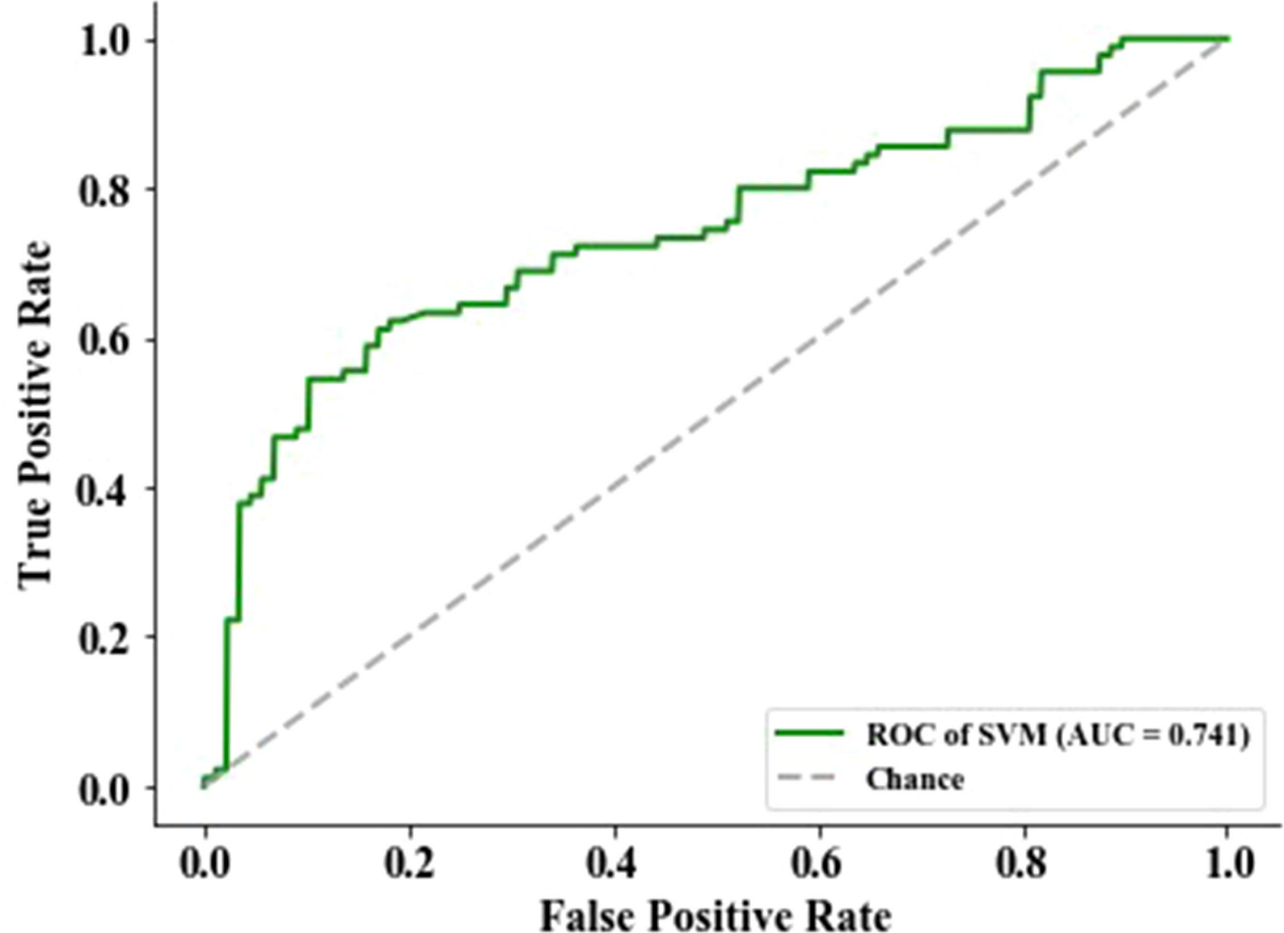
The performance of epidermal growth factor receptor (EGFR) status-related radiomics signature was evaluated by the area under the curve (AUC) of the receiver operating characteristic (ROC) curve.

The feature with a *p*-value >0.05 was excluded using the Mann–Whitney statistical test. Thus, the number of radiomics features was reduced from 2,153 to 1,545. Then, 13 normalized features with variance equal to zero were removed. The residual 1,532 features were sorted using mRMR algorithm to pick the 13 top-ranked features (34), including six (skewness, minimum, kurtosis, variance, minimum, and 10th percentile) in the Firstorder features that describe the distribution of voxel intensities within the image region defined by the mask through commonly used and basic metrics, one (SumSquares) in the gray-level co-occurrence matrix features that describe the second-order joint probability function of an image region constrained by the mask and is defined, three (SizeZoneNonUniformity, HighGrayLevelZoneEmphasis, and ZoneVariance) in the gray-level size zone matrix features that quantify the number of connected voxels sharing the same gray-level intensity in an image, and three (LargeDependenceHighGrayLevelEmphasis, LargeDependenceHighGrayLevelEmphasis, and DependenceEntropy) in the gray-level dependence matrix features that quantify the number of connected voxels within distance, which are dependent on the center voxel in an image. Then, the top-ranked features and four clinical features (age, gender, smoking, and tumor family history) were input into the SVM classifier to establish a radiomics signature that predicts EGFR mutation status in the training group (n = 514). The predictive score of the radiomics signature was assessed on an independent validation group with 178 patients and obtained an AUC of 74.13%, an F1 score of 68.29%, a specificity of 79.55%, an accuracy of 70.79%, and a sensitivity of 62.22% (**Figure 2**).

### Identification of the best response-related features based on 13 epidermal growth factor receptor mutant-associated features in epidermal growth factor receptor tyrosine kinase inhibitor therapy patients

To identify the imaging biomarkers candidates for the best response of EGFR TKI first-line treatment, the 13 top-rank radiomics features associated with EGFR mutation were further used to explore by logistic analysis. The two features that significantly negatively correlated with the best response were skewness (*p* = 0.004) and 10th percentile (*p* = 0.002) (**Table S1**). Skewness and 10th percentile were divided into two groups based on predicting the best response. ROC curve was applied to confirm the optimal cutoff points of significant features. For skewness and 10th percentile, the AUC values were 0.832 (*p* = 0.004, Youden’s index = 0.614) and 0.653 (*p* = 0.002, Youden’s index =0.289), respectively. The best cutoff points of skewness and 10th percentile, as confirmed by the AUC value, were 0.882 and 21.132, respectively.

**Figure 3:**
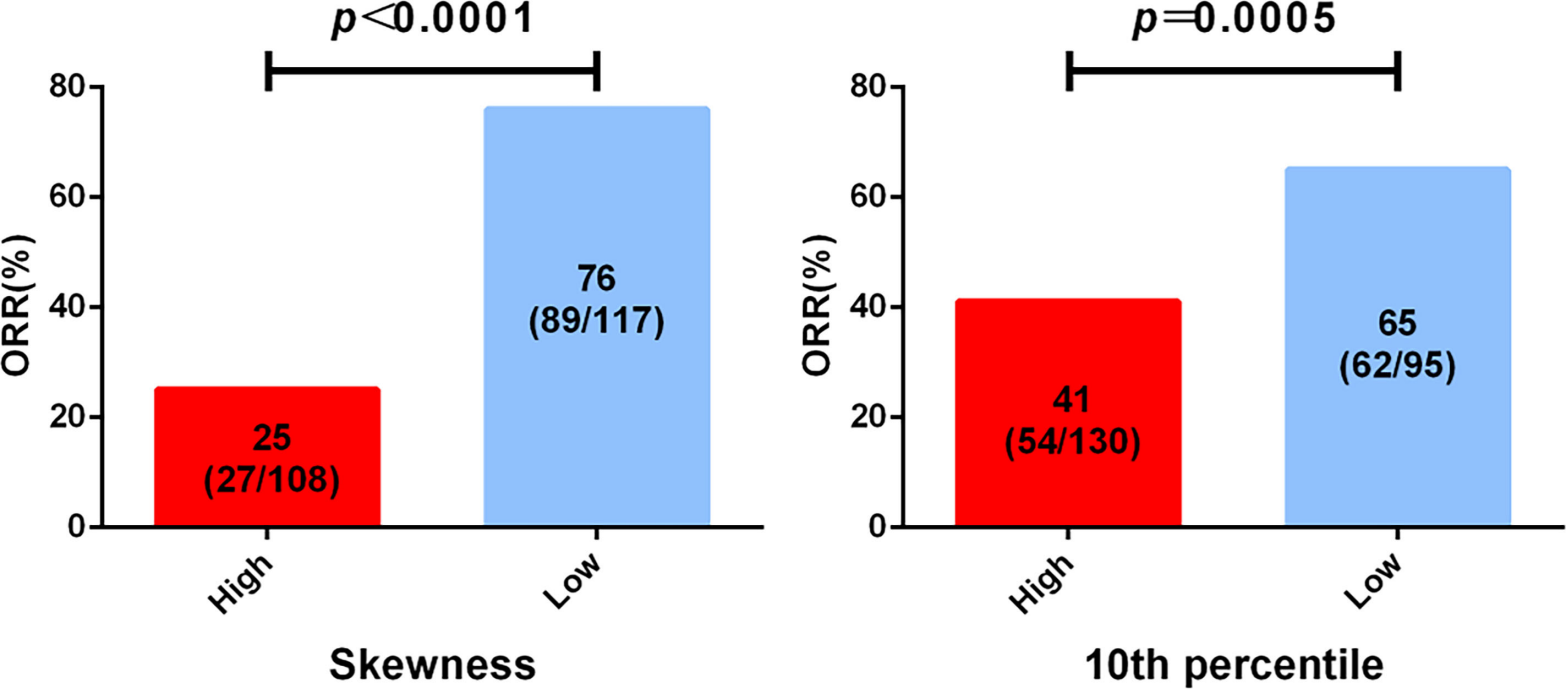
Analysis of epidermal growth factor receptor (EGFR) mutation-associated features from computed tomography (CT) imaging before treatment and the best clinical response to tyrosine kinase inhibitor (TKI) first-line therapy. All patients were divided into two groups according to the cutoff of skewness and 10th percentile.

Among the results from skewness, the skewness-L (≤0.882) group had a superior partial response (PR) rate than had the skewness-H (>0.882) group (89/117, 76.1% *vs* 27/108, 25.0%, HR = 9.536, 95% CI: 5.189–17.52, *p* < 0.0001) (**Figure 3**, **Table S2**). For the 10th percentile, the SD/PD rate was inferior in the 10th percentile-H group (>21.132) than in the 10th percentile-L group (≤21.132) (76/130, 58.5% *vs* 33/95, 34.7%, HR = 2.644, 95% CI: 1.529–4.574, *p* = 0.0005) (**Figure 3**, **Table S2**). In conclusion, we suggest that skewness and 10th percentile may be better predictive markers for differentiating response to first-line EGFR TKIs.

### Testing the correlation between the best response-associated features and progression-free survival

**Figure 4:**
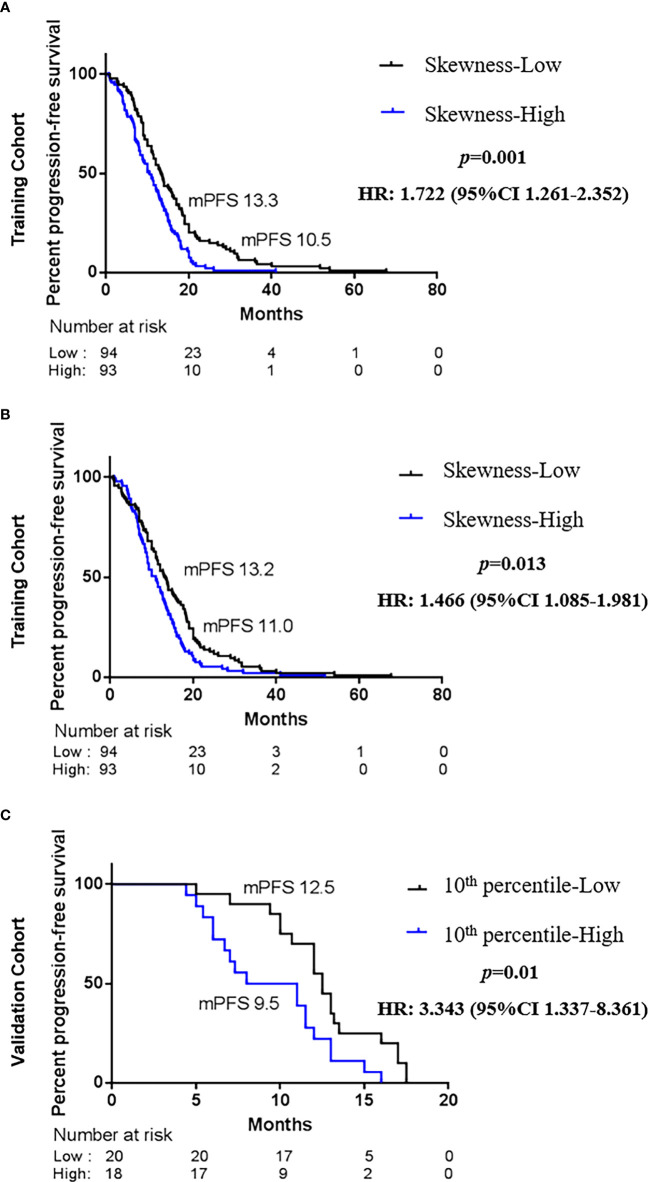
Kaplan–Maier survival curves of progression-free survival under biomarker-defined subgroups. In the tyrosine kinase inhibitor (TKI) therapy training cohort, **(A)** stratification by the skewness of first-order category (low ≤ 0.882 versus high > 0.882); **(B)** stratification by the 10th percentile of first-order category (low ≤ 21.132 versus high > 21.132). In the TKI therapy validation cohort, **(C)** stratification by the skewness of first-order category (low ≤ 0.882 versus high > 0.882). *p*-Values are calculated with multivariate Cox models adjusted by age, gender, smoking history, family history, TNM stage, and tumor position.

To explore whether advanced lung cancer patients with a good curative outcome can be distinct, we tested the defined cutoff points of the skewness of first-orders (≤0.882 versus >0.882) and the 10th percentile of first-orders (≤21.132 versus >21.132) in the training cohort (n = 187). Univariate analysis revealed that the skewness > 0.882 (*p* = 0.001) and the 10th percentile > 21.132 (*p* = 0.015) before treatment were associated with a significantly worse PFS. We then carried out a multivariate Cox proportional regression analysis containing these covariates to ensure independent factors. The relationship between two features and PFS was obvious in multivariate analysis; for the skewness, HR = 1.722, 95% CI: 1.261–2.352, *p* = 0.001 (**Figure 4A**, **Table 3**); for the 10th percentile, HR = 1.466, 95% CI: 1.085–1.981, *p* = 0.013 (**Figure 4B**, **Table 3**). Therefore, the skewness and 10th percentile of first-order features at baseline level could be used to predict the efficacy in EGFR-mutant advanced lung adenocarcinoma following standard first-line EGFR-TKI therapy.

**Table 3:** Multivariate analysis of the categorization of two features and PFS.

Factors	Categorization	Training cohort (*N*=187)		Validation cohort (*N*=38)	
		HR (95% CI)	*p*-Value	HR (95% CI)	*p*-Value
Age	Continuous	0.992 (0.976–1.009)	0.368	1.015 (0.976–1.055)	0.461
Gender	Female *vs* male	1.181 (0.751–1.856)	0.471	0.719 (0.154–3.360)	0.675
Smoking history	No *vs* yes	0.994 (0.609–1.621)	0.98	0.822 (0.180–3.744)	0.8
Family history	No *vs* yes	1.246 (0.764–2.031)	0.378	1.019 (0.357–2.907)	0.972
TNM stage^a^	III *vs* IV	0.854 (0.517–1.413)	0.54	0	0
Tumor position	RUL *vs* RML *vs* RLL *vs* LUL *vs* LLL	0.980 (0.887–1.083)	0.695	0.879 (0.671–1.153)	0.352
Skewness	≤Cutoff1^c^ *vs* >cutoff1^c^	1.722 (1.261–2.352)	0.001^b^	3.343 (1.337–8.361)	0.01^b^
Age	Male *vs* female	0.995 (0.979–1.012)	0.579	0.998 (0.961–1.036)	0.905
Gender	Continuous	1.154 (0.740–1.800)	0.528	1.694 (0.425–6.748)	0.455
Smoking history	0 or 1 *vs* 2	0.870 (0.534–1.416)	0.575	0.437 (0.109–1.751)	0.243
Family history	No *vs* yes	1.128 (0.694–1.833)	0.628	0.638 (0.237–1.718)	0.374
TNM stage^a^	First *vs* second	0.682 (0.418–1.111)	0.124	0	0
Tumor position	RUL *vs* RML *vs* RLL *vs* LUL *vs* LLL	0.996 (0.902–1.101)	0.943	1.013 (0.798–1.285)	0.919
10th percentile	≤Cutoff2^c^ *vs* >cut0ff2^c^	1.466 (1.085–1.981)	0.013^b^	1.122 (0.492–2.561)	0.784

PFS, progression-free survival; EGFR, epidermal growth factor receptor; TKI, tyrosine kinase inhibitor; RUL, right upper lung; RML, right middle lung; RLL, right lower lung; LUL, left upper lung; LLL, left lower lung; HR, hazard ratio; CI, confidence interval.

a Based on American Joint Committee on Cancer (AJCC) 8th edition.

b Only statistically significant (p < 0.05) results are reported for analysis.

c Cutoff1 = 0.882; Cutoff2 = 21.132.

### Validation of the predictive effect of skewness and 10th percentile features for progression-free survival

Next, we intended to validate the clinical effect of the skewness and 10th percentile of first-order features in the validation cohort (n = 38). Here, we used the aforesaid cutoff value in the training group: high skewness value (>0.882) versus low (≤0.882) and high 10th percentile value (>21.132) versus low (≤21.132). The correlation between the skewness of first-order and PFS is consistent with the training cohort (**Figure 4C**, **Table 3**). Probably due to the limited number of samples, we did not observe a statistical difference between the 10th percentile of first-order before treatment and PFS in the validation cohort (**Table 3**). However, the skewness of first-order is an effective biomarker that could better indicate the response of first-line EGFR-TKI therapy.

## Discussion

Therapeutic opportunities for EGFR mutant lung adenocarcinoma patients have radically changed because of the application of EGFR-TKI therapy. The response varies markedly, and more objective markers are needed to identify patients best suited for certain targeted therapies (4, 9, 15). EGFR mutation types are a well-studied biomarker of response to TKI therapy (6, 7, 36–41). Next-generation sequencing of tissue samples is the standard technique of EGFR status detection. Nevertheless, a biopsy is an invasive procedure of locating tissue that ignores organizational heterogeneity of tumor and microenvironment where distinct bioactive molecules can drive tumor development and progression.

Radiomics is a non-invasive technology that collects routine clinical medical images to assess the tumor phenotype (16, 17). Previous studies used clinical and radiomics models to predict EGFR mutation status (13, 14, 21–26). The radiomics features combined with the clinical factors had a greater prediction effect (13, 14, 22). For example, Aerts et al. found that homogeneity, inverse variance, sum entropy, short-run emphasis, maximum diameter, and tumor volume radiomics features had important roles in discriminating EGFR mutant status in lung adenocarcinoma (14). These features belong to GLCM, GLRLM, and shape features that reveal that EGFR mutation is more likely to be heterogeneous. Similarly, Ye et al., in a single group association study of lung adenocarcinomas, showed that CT imaging characteristics including bubble-like lucency and homogeneous enhancement were remarkably independent predictive factors for EGFR-activating mutation (22). The deep learning model also revealed that the deep learning features such as circle or arch shapes and horizontal and diagonal edges had a significant correlation between high-dimensional CT image characteristics and EGFR genotype (26). Based on previous research progress, we carried out new research for further exploration in this study, and we confirmed a radiomics signature by the SVM classifier combined with four clinical factors to forecast EGFR status in advanced lung adenocarcinoma using preoperative three-dimensional CT images. The radiomics signature showed strong predictive performance in the test group (AUC, 0.7413; specificity, 79.55%; accuracy, 70.79%). We found 13 radiological features that were remarkably associated with EGFR mutations; the first-order category had six features such as skewness, minimum, kurtosis, variance, and 10th percentile, which describes the distribution of voxel intensities within the image region defined by the mask through commonly used and basic metrics (34). The GLSZM and GLDM categories had three features each. The GLSZM is defined as the gray-level zone quantization of connected voxel numbers that share the same gray-level intensity in an image, while GLDM quantifies gray-level dependencies defined as the number of connected voxels within a distance that are dependent on the center voxel in an image. Together, the representation of these features indicates tumor heterogeneous related to EGFR mutation phenotype, which provides an alternative non-invasive way easily to forecast EGFR status in routine CT diagnosis and supply a good supplement to biopsy in real clinical practice.

Radiomics markers that can predict the efficacy of first-line EGFR-TKI therapy are now more needed; we also have found two CT features for progression risk stratification to first-line EGFR-TKI remedy in advanced lung adenocarcinoma. The skewness and 10th percentile of first-order features included in ROI preprocessed by gradient and exponential filter, respectively, were significantly negatively correlated with the best response and PFS of EGFR TKI therapy. Few studies used radiomics to explore the response of targeted therapy. For example, Jie Tian et al. extracted features from two-directional CT images and used the LASSO Cox regression analysis to select 12 CT features for discriminating between patients with rapid and slow progression to EGFR TKI therapy. The 12 CT features are part of GLCM, GLRLM, and first-order features (30). However, our study retrospectively obtained more comprehensive data of the tumors in 3D from CT images and was the first to further explore the relationship between EGFR mutation-related features and the response of EGFR TKI therapy in advanced lung adenocarcinoma. To date, several studies have found the first-order features with response and prognosis of EGFR TKI therapy, including energy, standard deviation, uniformity, and entropy (26, 30), but we are the first to find the predictive value of other first-order features for the best response and PFS. The skewness feature assesses the asymmetry of the distribution of values about the mean value, while the 10th percentile represents the first 10% proportion of voxels with positive order of susceptibility. The two features indicated the whole tumor heterogeneity and the asymmetry distribution of tumor parenchyma, which corresponds to the inhomogeneity of gross findings in CT images checked by the radiologist. It could explain why radiomics characteristics reveal treatment outcomes, while further work is needed to explore the potential mechanisms of the above features and predict the efficacy of lung cancer.

There are several limitations that could not be ignored. First, this was a retrospective study and CT images were acquired with 5-mm slice thicknesses, which is indeed used in hospitals. Although we may ignore some tumor information, our results are certainly closer to practice. Second, given that this was a single-center study, the study lacks an external validation group of patients, which is a key component of radiological analyses, and required validation in a larger patient population study. Third, our investigation only concentrated on EGFR mutation status. The interrelationship among radiomics features, EGFR, and other driver mutations (i.e., ROS-1, ALK, and c-Met) is unknown but could be studied in future research. Nonetheless, the study still had significant positive results. Further assessment of two indicators could be contained together with other predictive biomarkers in the evaluation of lung and other solid tumor patients who are candidates for treatment efficacy.

## Data availability statement

The raw data supporting the conclusions of this article will be made available by the authors, without undue reservation.

## Ethics statement

Ethical review and approval was not required for the study of human participants in accordance with the local legislation and institutional requirements. Written informed consent from the patients/participants was not required to participate in this study in accordance with the national legislation and the institutional requirements.

## Author contributions

Conception and design: LW, MJ, PY, and JiL. Acquisition of data (acquired and managed patients): MJ, PY, JiL, WP, XP, BC, JaL, and JW. Analysis and interpretation of data: MJ and PY. Writing, review, and/or revision of the manuscript: MJ, PY, and JiL. LW designed and supervised the study. All authors contributed to the article and approved the submitted version.

## Funding

This work was supported by the Hunan Provincial Natural Science Foundation of China (No. 2021JJ30430), Wu Jieping Medical Foundation (No. 320.6750.19088-11) and the Research Foundation of Chinese Sociaty of Clinical Oncology (No. Y-2019Genecast-024 and No. Y-HR2019-0464) to LW. This work was supported by the National Cancer Center (NCC2017A17) to PY.

## Acknowledgments

We thank all patients, family, nurses, and doctors for their contribution to this study.

## Conflict of interest

The authors declare that the research was conducted in the absence of any commercial or financial relationships that could be construed as a potential conflict of interest.

## Publisher’s note

All claims expressed in this article are solely those of the authors and do not necessarily represent those of their affiliated organizations, or those of the publisher, the editors and the reviewers. Any product that may be evaluated in this article, or claim that may be made by its manufacturer, is not guaranteed or endorsed by the publisher.
