# Supplementary material for: Computed tomography-based radiomics quantification predicts epidermal growth factor receptor mutation status and efficacy of first-line targeted therapy in lung adenocarcinoma
Source: Front Oncol. 2022 Aug 16;12:985284. doi: 10.3389/fonc.2022.985284 (PMC9424619; doi:10.3389/fonc.2022.985284)
Supplement: Supplementary file 1 [file Table_1.docx]

| Table S1. Analysis of variables and best response in EGFR-TKI therapy cohort | | | |  |
| --- | --- | --- | --- | --- |
| Features | category | preprocessed filter | *p*-value |  |
|  |  |  |  |  |
| **Skewness** | Firstorder | gradient | 0.004^a^ |  |
| **Minimum** | Firstorder | wavelet-LHL | 0.862 |  |
| **Kurtosis** | Firstorder | wavelet-HHL | 0.069 |  |
| **Variance** | Firstorder | exponential | 0.761 |  |
| **Minimum** | Firstorder | log-sigma-3-0-mm-3D | 0.534 |  |
| **10th Percentile** | Firstorder | exponential | 0.002^a^ |  |
| **SumSquares** | GLCM | wavelet-LHL | 0.107 |  |
| **SizeZoneNonUniformity** | GLSZM | squareroot | 0.106 |  |
| **HighGrayLevelZoneEmphasis** | GLSZM | wavelet-LLH | 0.825 |  |
| **ZoneVariance** | GLSZM | original | 0.115 |  |
| **LargeDependence**  **HighGrayLevelEmphasis** | GLDM | wavelet-HLH | 0.223 |  |
| **LargeDependence**  **HighGrayLevelEmphasis** | GLDM | wavelet-LHH | 0.401 |  |
| **DependenceEntropy** | GLDM | wavelet-LLH | 0.370 |  |

^a^Only statistically significant (*p*<0.05) results are reported for analysis.

Abbreviations: EGFR, epidermal growth factor receptor; GLCM, gray level co-occurrence matrix; GLDM, gray level dependence matrix; GLSZM, gray level size zone matrix; support vector machine; TKI, tyrosine kinase inhibitor。
