# Supplementary material for: Computed tomography-based radiomics quantification predicts epidermal growth factor receptor mutation status and efficacy of first-line targeted therapy in lung adenocarcinoma
Source: Front Oncol. 2022 Aug 16;12:985284. doi: 10.3389/fonc.2022.985284 (PMC9424619; doi:10.3389/fonc.2022.985284)
Supplement: Supplementary file 2 [file Table_2.docx]

| Table S2. Radiomic features and the best clinical response in the total cohort | | | | |
| --- | --- | --- | --- | --- |
| Parameters | Patients with response data(N=225) | | | |
|  | PR | SD-PD | HR (95%CI) | *p*-value |
| skewness-H (＞0.882) | 27 | 81 | 9.536(5.189-17.52) | <0.0001^a^ |
| skewness-L (≤0.882) | 89 | 28 |  |  |
| 10^th^ percentile-H (＞21.132) | 54 | 76 | 2.644(1.529-4.574) | 0.0005^a^ |
| 10^th^ percentile-L (≤21.132) | 62 | 33 |  |  |

^a^Only statistically significant (*p*<0.05) results are reported for analysis.

Abbreviations: PR, partial response; SD, stable disease; PD, progressive disease; HR, hazard ratio; CI, confidence interval; H, high; L, low.
